# Analysis of body composition characteristics in patients with type 1 diabetes mellitus: a case–control study based on a bioelectrical impedance analyzer

**DOI:** 10.3389/fmed.2026.1776074

**Published:** 2026-04-21

**Authors:** Lun Zhang, Jiani Zhu, Ran Gu, Tongfen Cui, Xiaoyun Xi, Luying Yang, Guang Yu, Ji-Gan Wang, Fan Yang

**Affiliations:** 1 Department of Clinical Nutrition, The First People’s Hospital of Yunnan Province, The Affiliated Hospital of Kunming University of Science and Technology, Kunming, Yunnan, China; 2 Department of General Medicine, Buga Town Health Center, Zhaotong, Yunnan, China; 3 Department of Endocrine Metabolism, Changning County People’s Hospital, Baoshan, Yunnan, China; 4 Department of Clinical Nutrition, Baoshan Second People’s Hospital, Baoshan, Yunnan, China; 5 Department of Endocrine Metabolism, Menghai County People’s Hospital, Xishuangbanna, Yunnan, China; 6 Department of Pediatrics, Maternal and Child Health Hospital of Guangxi Zhuang Autonomous Region, Nanning, China

**Keywords:** BIA, BMI, body composition analysis, SMI, T1DM

## Abstract

**Objective:**

This study aimed to systematically compare differences in body composition between adult patients with type 1 diabetes mellitus (T1DM) and healthy controls using the bioelectrical impedance analysis (BIA) method, to provide an objective basis for clinical nutritional assessment and intervention in T1DM patients.

**Methods:**

A total of 57 T1DM patients hospitalized at the First People’s Hospital of Yunnan Province between December 2023 and April 2024 were selected as the study group, and 55 healthy adults matched for age, sex, and BMI were recruited as the control group. Body composition indicators, including body fat, fat-free mass, skeletal muscle, visceral fat area, and phase angle (PA), were measured for all subjects using a body composition analyzer, and the skeletal muscle mass index (SMI) was calculated. BIA was used to determine body composition parameters, and statistical analyses were conducted to compare the differences between the two groups.

**Results:**

In males, the T1DM group exhibited significantly lower body fat, body fat percentage, bone mineral content, visceral fat area, and SMI compared to the healthy control group (all *p* < 0.05). In females, the T1DM group showed significantly higher levels of intracellular water, protein, minerals, skeletal muscle, fat-free mass, right leg lean mass, basal metabolic rate, and SMI, while demonstrating significantly lower body fat, body fat percentage, visceral fat area, and waist-to-hip ratio compared to the control group (all *p* < 0.05).

**Conclusion:**

Despite having normal or even low BMI, T1DM patients present a unique body composition profile characterized by “low fat mass, low visceral fat, but relatively high skeletal muscle mass.” This suggests that the disease state may influence energy metabolism and body composition.

## 1 Introduction

Diabetes mellitus represents a major global public health challenge. Among its forms, type 1 diabetes mellitus (T1DM) is a chronic metabolic disease characterized by autoimmune-mediated destruction of pancreatic beta cells, leading to an absolute deficiency of insulin (1–3). Unlike type 2 diabetes, T1DM typically manifests at a younger age and is often associated with a leaner body build, with its metabolic dysregulation and management strategies being distinct (4–6). For a long time, body mass index (BMI) has been widely used to assess an individual’s nutritional status and health risks. However, as a general indicator, BMI cannot distinguish between fat mass and fat-free mass in the body, nor can it reflect the distribution of body fat (such as visceral fat accumulation) or changes in body cell mass (7, 8). This limitation is particularly significant in patients with T1DM: although many patients present with a normal or low BMI, they may harbor underlying alterations in body composition detrimental to metabolic health, such as abnormal adipose tissue proportions, reduced muscle mass, or visceral fat accumulation. This “hidden” abnormality in body composition is likely interrelated with various factors specific to T1DM, including long-term glycemic fluctuations, exogenous insulin therapy, and alterations in energy metabolism (9).

Current research on body composition in patients with type 1 diabetes mellitus (T1DM) remains controversial. Some studies indicate that, due to impaired insulin-mediated anabolic effects and increased gluconeogenesis, T1DM patients may be at risk of muscle mass loss (10). Conversely, other studies have observed an elevated body fat percentage, particularly visceral fat accumulation, in some patient cohorts (11). These inconsistent findings suggest that the impact of T1DM on body composition is likely a complex, multifactorial process, warranting more systematic and detailed evaluation.

Bioelectrical impedance analysis (BIA) is a non-invasive, convenient, and highly reproducible method for assessing body composition and has been widely adopted in both clinical and research settings (12, 13). Its derived parameter, the phase angle (PA), is recognized as a potential indicator of cellular membrane integrity and body cell mass, demonstrating significant value in nutritional and prognostic assessment across various disease states (14). However, there is currently a scarcity of studies that systematically utilize BIA technology to investigate the body composition characteristics of Chinese adult T1DM patients while employing rigorously matched healthy controls.

Therefore, this study aims to employ the BIA method within a carefully designed case–control framework. By strictly controlling for confounding factors such as age, sex, and BMI, we seek to systematically compare differences in overall and regional body composition between T1DM patients and healthy adults. The objective is to elucidate the distinct body composition alteration patterns associated with T1DM, thereby providing an objective basis for conducting precise nutritional assessments and developing individualized nutritional and exercise intervention strategies in clinical practice.

## 2 Materials and methods

### 2.1 Study participants

According to the inclusion criteria, 57 patients with type 1 diabetes mellitus (T1DM) hospitalized in the Department of Endocrinology and Metabolism at the First People’s Hospital of Yunnan Province between December 2023 and April 2024 were recruited as the study group. For the control group, 55 healthy adults were matched (frequency matching) based on age, sex, and BMI. These controls were selected from individuals receiving nutritional counseling in the Department of Clinical Nutrition at the same hospital during the same period.

Inclusion criteria for the T1DM group were as follows:

① Met the diagnostic criteria for T1DM as outlined in the “Chinese Guidelines for the Diagnosis and Treatment of Type 1 Diabetes Mellitus (2021 Edition)” (15).
② Were conscious, had no communication barriers, and were able to cooperate with the study measurements and physical assessments.
③ Aged between 18 and 70 years.
④ Had no prior history of gastrointestinal diseases or digestive tract surgery.
⑤ Had a disease duration of not less than 1 year.

Exclusion criteria (applied to both groups) were:

① Inability to cooperate and complete the required examinations.
② Pregnancy or lactation in females.
③ Long-term use of medications such as thyroid drugs, steroids, or estrogen/progestin.
④ Presence of implanted medical devices including cardiac pacemakers, implantable cardioverter-defibrillators, or prosthetic joints.

All participants provided informed consent, and this study was approved by the Ethics Committee of the First People’s Hospital of Yunnan Province (Ethics Approval No. KHLL2022-KY165).

### 2.2 Methods

#### 2.2.1 Body composition measurement

Body composition of participants in both groups was assessed using a bioelectrical impedance body composition analyzer (Donghuayuan, Model 610). The specific operational procedures were as follows:

Prior to measurement, subjects were required to maintain a fasting state and evacuate their bowels and bladder. It was reconfirmed that no participants had cardiac pacemakers, other electronic medical devices, or metal implants (such as steel plates or pins) that could interfere with the measurement. The electrode cables were connected to the analyzer, and the power for both the printer and the main instrument was switched on. Subjects were instructed to remove their shoes, socks, and any metal objects, ensuring full exposure of the sites for electrode contact. The electrode contact areas on the hands and feet, as well as the electrodes themselves, were cleaned with moist towelettes. Subject information was then accurately entered into the system as prompted by the operation interface.

#### 2.2.2 Measurement process

The subject’s arms were appropriately abducted at a 15-degree angle from the torso, and feet were separated shoulder-width apart. Electrodes were connected to the four limbs as required. Press the “Enter” key to access the operation interface; during the measurement, the screen displays the progress, and the measurement is completed when the progress reaches 100%. After measurement, remove the electrodes and print the report sheet. Directly use the values from the report sheet, including intracellular water, extracellular water, protein, inorganic salts, body fat, body weight, height, skeletal muscle, body fat percentage, body mass index (BMI), body cell mass, bone mineral content, upper arm circumference, upper arm muscle circumference, waist circumference, visceral fat area, basal metabolic rate, and the phase angle (PA) value at 50 kHz frequency, and calculate the skeletal muscle mass index (SMI).

### 2.3 Statistical methods

Data processing and analysis were performed using SPSS software (version 26.0). Continuous data conforming to a normal distribution are presented as the mean (±standard deviation) and were compared between groups using the independent samples t-test. Continuous data not conforming to a normal distribution are presented as the median (25th percentile, 75th percentile) [M (P25, P75)] and were compared between groups using non-parametric tests. Categorical data are expressed as frequencies and percentages (%), and comparisons between groups were made using the chi-square test. A *p*-value of less than 0.05 (*p* < 0.05) was considered statistically significant.

## 3 Results

### 3.1 Comparison of age and BMI between the two groups

A total of 57 participants were ultimately enrolled in the T1DM group, and 55 in the healthy control group. The T1DM group comprised 20 males and 37 females, while the control group consisted of 18 males and 37 females. Among males, age in the T1DM group and the healthy control group was 37.90 ± 15.64 years and 37.89 ± 15.60 years, respectively, and BMI was 21.06 ± 3.52 kg/m^2^ and 21.72 ± 3.57 kg/m^2^, respectively. No statistically significant differences were found in age or BMI between the two groups (*p* > 0.05). Among females, age in the T1DM group and the healthy control group was 45.00 (21.00, 51.50) years and 44.00 (23.50, 53.00) years, respectively, and BMI was 20.22 ± 2.77 kg/m^2^ and 21.06 ± 3.52 kg/m^2^, respectively. No statistically significant differences were found in age or BMI between the two groups (*p* > 0.05) (see Table 1).

**Table 1:** Comparison of age and BMI between the two groups.

Indicator	Male		Female	
	T1DM (*n* = 20)	Control group (*n* = 18)	T1DM (*n* = 37)	Control group (*n* = 37)
Age (years)	37.90 ± 15.64	37.89 ± 15.60	45.00 (21.00, 51.50)	44.00 (23.50, 53.00)
BMI (kg/m^2^)	21.06 ± 3.52	21.72 ± 3.57	20.22 ± 2.77	20.45 ± 2.57
	*p* > 0.05		*p* > 0.05	

### 3.2 Comparison of results by BMI weight status between groups

Among males, no statistically significant difference was found in the distribution of body types between the T1DM group and the healthy control group (*p* > 0.05). Similarly, among females, no statistically significant difference was observed in the distribution of body types between the two groups (*p* > 0.05) (see Table 2).

**Table 2:** Comparison of body type outcomes between the two groups [*n* (%)].

Body type	Male		Female	
	T1DM	Control group	T1DM	Control group
Underweight	4 (66.67)	2 (33.33)	11 (55.00)	9 (45.00)
Normal (weight)	12 (50.00)	12 (50.00)	22 (47.83)	24 (52.17)
Overweigh	3 (50.00)	3 (50.00)	4 (50.00)	4 (50.00)
Obesity	1 (50.00)	1 (50.00)	0	0
*χ* ^2^	0.574		0.287	
*p*	0.905		0.866	

### 3.3 Comparison of body composition between the two groups (males)

In males, body fat mass was 7.05 (4.52, 14.15) kg in the T1DM group versus 12.15 (9.00, 18.27) kg in the healthy control group; body fat percentage was 13.68 ± 8.63% versus 21.30 ± 7.62%; bone mineral content was 2.83 ± 0.36 kg versus 3.15 ± 0.37 kg; visceral fat area was 38.88 ± 26.49 cm^2^ versus 59.02 ± 33.16 cm^2^; and skeletal muscle mass index (SMI) was 7.93 (7.28, 8.34) kg/m^2^ versus 7.19 (6.65, 7.74) kg/m^2^. These differences were statistically significant (*p* < 0.05) (see Table 3).

**Table 3:** Comparison of body composition results between the two groups (male).

Indicator	T1DM	Control group	*T*/*Z*	*p*
Phase angle (°)	5.66 ± 1.20	5.83 ± 0.61	−0.566	0.576
ECW (L)	23.80 (21.92, 25.17)	22.60 (19.87, 24.55)	−1.141	0.254
ICW (L)	14.60 (13.75, 15.22)	13.80 (12.35, 15.22)	−1.347	0.178
Protein (kg)	10.30 (9.50, 10.87)	9.75 (8.57, 10.60)	−1.185	0.236
Mineral (kg)	3.54 (3.19, 3.65)	3.49 (2.98, 3.83)	−0.307	0.759
Body fat (kg)	7.05 (4.52, 14.15)	12.15 (9.00, 18.27)	−2.442	0.015
FFM (kg)	51.45 (47.62, 54.52)	49.50 (43.82, 54.32)	−0.877	0.380
Skeletal muscle (kg)	29.00 (25.67, 30.85)	27.45 (23.87, 30.02)	−0.096	0.365
LBM (kg)	47.93 (44.43, 50.85)	45.99 (43.82, 54.32)	−0.950	0.342
Body fat percentage (%)	13.68 ± 8.63	21.30 ± 7.62	−2.868	0.007
Right arm muscle mass (kg)	2.79 (2.37, 2.94)	2.55 (2.27, 2.84)	−0.789	0.430
Left arm muscle mass (kg)	2.70 (2.35, 2.89)	2.43 (2.19, 2.77)	−0.863	0.388
Trunk muscle mass (kg)	22.65 (20.57, 23.65)	22.25 (19.55, 24.23)	−0.029	0.977
Right leg lean mass (kg)	8.38 ± 1.60	7.88 ± 0.84	1.185	0.244
Left leg lean mass (kg)	8.25 ± 1.47	7.87 ± 0.83	0.963	0.342
BMC (kg)	2.83 ± 0.36	3.15 ± 0.37	−2.663	0.012
VAT (cm^2^)	38.88 ± 26.49	59.02 ± 33.16	−2.078	0.045
BMR (kcal)	1498.00 (1432.25, 1547.50)	1438.65 (1316.32, 1542.95)	−1.802	0.279
SMI (kg/m^2^)	7.93 (7.28, 8.34)	7.19 (6.65, 7.74)	−2.397	0.017
WHR	0.88 ± 0.06	0.85 ± 0.05	1.907	0.065

### 3.4 Comparison of body composition results between the two groups (females)

In female subjects, intracellular water (L) was 18.15 ± 2.18 in the T1DM group and 16.75 ± 2.14 in the healthy control group; extracellular water (L) was 11.10 (10.40, 12.10) and 14.90 (11.80, 17.45); protein (kg) was 7.85 ± 0.94 and 7.23 ± 0.92; minerals (kg) was 2.95 ± 0.33 and 2.67 ± 0.31; body fat (kg) was 11.10 (6.65, 14.85) and 14.90 (11.80, 17.45); fat-free mass (kg) was 40.30 (36.10, 43.30) and 37.30 (34.60, 39.85); skeletal muscle (kg) was 21.50 ± 2.90 and 19.85 ± 2.80; lean body mass (kg) was 37.51 (33.37, 39.98) and 34.57 (32.10, 37.04); body fat percentage (%) was 19.95 ± 8.07 and 28.25 ± 4.70; right leg muscle mass (kg) was 6.51 (5.65, 7.17) and 5.94 (5.51, 6.51); visceral fat area (cm^2^) was 42.50 (30.80, 63.85) and 63.30 (47.80, 79.95); basal metabolic rate (kcal) was 1247.00 (1156.00, 1304.85) and 1174.80 (1117.25, 1230.50); SMI (kg/m^2^) was 6.24 (5.47, 0.73) and 5.94 (5.53, 6.28); waist-to-hip ratio was 0.85 (0.79, 0.91) and 0.82 (0.79, 0.84). The differences were statistically significant (*p* < 0.05) (see Table 4).

**Table 4:** Comparison of body composition results between the two groups (female).

Indicator	T1DM	Control group	*T*/*Z*	*p*
Phase angle (°)	5.00 (4.65, 5.50)	4.91 (4.67, 5.26)	−0.773	0.439
ECW (L)	18.15 ± 2.18	16.75 ± 2.14	2.766	0.007
ICW (L)	11.10 (10.40, 12.10)	14.90 (11.80, 17.45)	−2.429	0.015
Protein (kg)	7.85 ± 0.94	7.23 ± 0.92	2.821	0.006
Mineral (kg)	2.95 ± 0.33	2.67 ± 0.31	3.655	<0.001
Body fat (kg)	11.10 (6.65, 14.85)	14.90 (11.80, 17.45)	−2.935	0.003
FFM (kg)	40.30 (36.10, 43.30)	37.30 (34.60, 39.85)	−2.525	0.012
Skeletal muscle (kg)	21.50 ± 2.90	19.85 ± 2.80	2.488	0.015
LBM (kg)	37.51 (33.37, 39.98)	34.57 (32.10, 37.04)	−2.427	0.015
Body fat percentage (%)	19.95 ± 8.07	28.25 ± 4.70	−5.045	<0.001
Right arm muscle mass (kg)	1.71 (1.46, 2.09)	1.63 (1.46, 1.85)	−0.995	0.320
Left arm muscle mass (kg)	1.64 (1.49, 1.85)	1.61 (1.41, 1.80)	−1.152	0.249
Trunk muscle mass (kg)	16.71 ± 2.32	16.63 ± 2.05	0.167	0.867
Right leg lean mass (kg)	6.51 (5.65, 7.17)	5.94 (5.51, 6.51)	−2.011	0.004
Left leg lean mass (kg)	6.23 (5.43, 6.94)	5.96 (5.56, 6.48)	−1.378	0.168
BMC (kg)	2.53 ± 0.36	2.46 ± 0.29	0.860	0.393
VAT (cm^2^)	42.50 (30.80, 63.85)	63.30 (47.80, 79.95)	−3.687	<0.001
BMR (kcal)	1247.00 (1156.00, 1304.85)	1174.80 (1117.25, 1230.50)	−2.685	0.004
SMI (kg/m^2^)	6.24 (5.47, 6.73)	5.94 (5.53, 6.28)	−2.005	0.045
WHR	0.85 (0.79, 0.91)	0.82 (0.79, 0.84)	−2.272	0.023

ECW, extracellular water; ICW, intracellular water; FFM, fat-free mass; LBM, lean body mass; VAT, visceral adipose tissue; BMC, bone mineral content; BMR, basal metabolic rate; WHR, waist-to-hip ratio; SMI, skeletal muscle index.

## 4 Discussion

This study employed bioelectrical impedance analysis to systematically compare body composition differences between T1DM patients and healthy adults under conditions matched for age, sex, and BMI. The main results revealed a distinct body composition profile in T1DM patients: despite comparable overall body weight and BMI with the healthy population, they exhibited significantly lower body fat mass, body fat percentage, and visceral fat area, while demonstrating significantly higher fat-free mass, protein content, and SMI, an indicator reflecting muscle mass. This finding of “lower fat but higher muscle” provides new insights for understanding the metabolic characteristics of T1DM.

The results of this study demonstrate that in the male T1DM group, body fat mass, body fat percentage, bone mineral content, and visceral fat area were all lower than those in the healthy control group, while the SMI was significantly higher (*p* < 0.05). In the female T1DM group, intracellular water, protein, minerals, fat-free mass, skeletal muscle mass, lean body mass, and SMI were all higher than those in the healthy control group, while extracellular water, body fat mass, body fat percentage, and visceral fat area were lower (*p* < 0.05). This indicates that both male and female T1DM patients exhibited lower body fat mass, body fat percentage, and visceral fat area, which is consistent with the findings reported by Ingberg et al. (16). Additionally, Paulino et al. (17) reported that pediatric T1DM patients also had lower body fat levels. However, the conclusions of this study differ from some other research reports. Some studies have shown that adult T1DM patients have a higher prevalence of overweight and obesity compared to healthy adults, particularly with females exhibiting higher fat mass in both limbs and trunk (18). Furthermore, Nsamba et al. (19) found that children with diabetes showed more pronounced features of obesity compared to the control group, and abdominal fat accumulation was closely associated with poorer glycemic control. The discrepancy between these study findings may be attributed to the BMI matching employed in the participant recruitment process of this study.

Most patients with type 2 diabetes mellitus (T2DM) present with symptoms of sarcopenia (20, 21). In contrast, this study found relatively higher muscle mass in T1DM patients—a finding that diverges from T2DM research outcomes yet highlights the distinct pathophysiological mechanism of T1DM. Potential explanations include: firstly, under conditions of absolute insulin deficiency, lipoprotein lipase activity is suppressed, impairing fat synthesis and storage while enhancing lipolysis, thereby promoting fat mobilization (22). Secondly, chronic hyperglycemia leads to increased urinary glucose excretion, which represents a form of energy loss, shifting overall energy balance toward a negative state unfavorable for fat accumulation (4). Additionally, some patients may intentionally restrict dietary intake due to concerns about glycemic fluctuations, which may further limit energy consumption. Our findings suggest that when assessing metabolic risk in T1DM patients, the “obesity-centered” paradigm based on T2DM should not be directly applied, as a low-fat state may be a characteristic manifestation in a subset of T1DM patients.

Another noteworthy finding worthy of in-depth discussion is that patients in the T1DM group exhibited higher SMI, protein content, and fat-free mass compared to the control group. Conventional understanding suggests that insulin deficiency and enhanced gluconeogenesis may lead to muscle protein catabolism. However, the contrary results observed in this study may stem from the following factors: Firstly, exogenous insulin therapy, particularly the basal-bolus insulin regimen, partially restores anabolic signaling, potentially promoting the maintenance or even growth of muscle tissue (23). Secondly, for glycemic control purposes, patients might engage in more conscious physical activity compared to the general healthy population, and regular exercise stimulation helps preserve muscle mass (24). Thirdly, the possibility of “survivorship bias” cannot be excluded, whereby patients with longer disease duration and stable conditions, who likely possess stronger self-management capabilities, maintain a more favorable body composition.

### 4.1 The “high muscle mass and low bone mineral” phenomenon

In the male T1DM group, SMI was significantly higher than in the control group, yet the bone mineral content was significantly lower (2.83 kg vs. 3.15 kg, *p* = 0.012), presenting a dissociation between high muscle mass and low bone mass. This suggests that under the chronic condition of insulin deficiency supplemented by exogenous insulin, osteoblast function remains suppressed, potentially related to chronic hyperglycemia, decreased insulin-like growth factor-1 (IGF-1), increased urinary calcium loss, and low-grade inflammation (25). Although no statistically significant difference was observed in females, the absolute value of bone mineral content was also lower than in the control group, further supporting the risk of reduced bone mass in T1DM (26, 27). Clinical practice should prioritize early bone density screening, along with ensuring adequate calcium and vitamin D intake and implementing resistance exercise to prevent fragility fractures. However, this phenomenon is not entirely consistent with the current general understanding. Therefore, we approach it with caution and prefer to regard it as an association observed in the specific sample of this study, rather than as an inherent physiological characteristic of type 1 diabetes. Considering the potential for selection bias (for example, the enrolled patients may have had stronger health management awareness) as well as the technical limitations of bioelectrical impedance analysis, which is influenced by hydration status, this finding still requires validation in broader populations using more rigorous adjustment methods.

Phase angle (PA) is an important parameter in bioelectrical impedance analysis. It is calculated from resistance and reactance, and can reflect cell membrane integrity, cellular functional status, and the nutritional condition of the body (28). In general, a higher phase angle indicates better-preserved cellular structure and higher body cell mass, whereas a lower phase angle may be associated with inflammation, malnutrition, and the progression of chronic diseases (29). In the field of diabetes research, some studies have found that patients with type 2 diabetes have lower phase angle values than healthy individuals, and that these values are associated with insulin resistance, chronic inflammation, and metabolic abnormalities (30). However, studies on phase angle changes in patients with type 1 diabetes remain limited. Although phase angle was measured in this study, no statistically significant difference was observed between the case group and the control group. This result may suggest that, in patients with relatively stable type 1 diabetes, cell membrane integrity and body cell mass are not markedly impaired, or that the body maintains a relatively stable state of cellular function through long-term metabolic adaptation. In addition, phase angle is influenced by multiple factors, including age, sex, body composition, and fluid distribution (31). The sample size of this study was relatively limited, and there was a certain degree of heterogeneity among participants in terms of disease duration, glycemic control, and insulin treatment regimens, all of which may have weakened the statistical detectability of intergroup differences.

### 4.2 Clinical implications and significance of the findings

The findings of this study carry important clinical implications. They underscore that relying solely on BMI for nutritional assessment and health risk stratification is far from sufficient for T1DM patients. Clinicians should move beyond BMI and utilize tools like body composition analysis to gain a more precise understanding of a patient’s body composition. For patients exhibiting the “low fat, high muscle” profile, the focus of nutritional support may not be traditional “weight loss” but should instead emphasize: (1) ensuring adequate energy and protein intake to maintain and even optimize existing muscle mass, thereby preventing malnutrition; (2) optimizing the quality of fat intake to guarantee the supply of essential fatty acids and fat-soluble vitamins; and (3) developing individualized exercise prescriptions to preserve muscle mass while further improving insulin sensitivity and overall metabolic health.

This study also has several limitations. First, although bioelectrical impedance analysis is convenient to perform, its results are easily influenced by the body’s hydration status. Although participants in this study were required to undergo measurements in the fasting state to minimize interference as much as possible, the use of bioelectrical impedance analysis for body composition assessment still has inherent limitations. This method is highly sensitive to hydration status, and in patients with type 1 diabetes, glycemic fluctuations, acute hyperglycemia, or ketosis may significantly affect fluid balance. In this study, key information was not collected prior to measurement, including recent glycemic control status (such as time in range), real-time blood glucose levels, ketone data, the timing of the last insulin administration, and exclusion of menstrual cycle phase data in females. The absence of these data may have led to biased estimates of parameters such as fat-free mass, intracellular/extracellular fluid distribution, and phase angle. Second, this study did not include or adjust for several important diabetes-specific variables, including detailed indicators of glycemic control (such as HbA1c), exact disease duration, insulin treatment regimen and dosage, chronic complications, and physical activity level. All of these factors have significant effects on body composition, including muscle mass and fat distribution. Their omission may have introduced confounding bias, making it difficult to attribute the observed differences entirely to the “disease state” of type 1 diabetes itself. Third, the sample size of this study was relatively small (*n* = 112), and the statistical power was further reduced after subgroup analyses by sex, which may have increased the risk of type II error, particularly for indicators such as phase angle and bone mineral content in the female subgroup. Therefore, some negative findings should be interpreted with caution, and future studies should expand the sample size and perform power calculations to verify these conclusions.

Future studies may employ more precise methods such as dual-energy X-ray absorptiometry (DXA) or magnetic resonance imaging (MRI) to measure body composition, and compare them with the INBODY method. Additionally, comprehensive assessment data related to diseases, such as insulin injection regimens, glycemic control status, exercise status, and seasonal factors, should be improved. Furthermore, the relationship between dietary habits and body composition and changes can be explored to further validate the conclusions of this study.

## Data Availability

The original contributions presented in the study are included in the article/supplementary material, further inquiries can be directed to the corresponding author.
